# Reduced Fitness of Virulent *Aphis glycines* (Hemiptera: Aphididae) Biotypes May Influence the Longevity of Resistance Genes in Soybean

**DOI:** 10.1371/journal.pone.0138252

**Published:** 2015-09-15

**Authors:** Adam J. Varenhorst, Michael T. McCarville, Matthew E. O’Neal

**Affiliations:** Department of Entomology, Iowa State University, Ames, IA 50011, United States of America; Agriculture and Agri-Food Canada, CANADA

## Abstract

Sustainable use of insect resistance in crops require insect resistance management plans that may include a refuge to limit the spread of virulence to this resistance. However, without a loss of fitness associated with virulence, a refuge may not prevent virulence from becoming fixed within a population of parthenogenetically reproducing insects like aphids. Aphid-resistance in soybeans (*i*.*e*., *Rag* genes) prevent outbreaks of soybean aphid (*Aphis glycines*), yet four biotypes defined by their capacity to survive on aphid-resistant soybeans (*e*.*g*., biotype-2 survives on *Rag1* soybean) are found in North America. Although fitness costs are reported for biotype-3 on aphid susceptible and *Rag1* soybean, it is not clear if virulence to aphid resistance in general is associated with a decrease in fitness on aphid susceptible soybeans. In laboratory assays, we measured fitness costs for biotype 2, 3 and 4 on an aphid-susceptible soybean cultivar. In addition, we also observed negative cross-resistance for biotype-2 on *Rag3*, and biotype-3 on *Rag1* soybean. We utilized a simple deterministic, single-locus, four compartment genetic model to account for the impact of these findings on the frequency of virulence alleles. When a refuge of aphid susceptible was included within this model, fitness costs and negative cross-resistance delayed the increase of virulence alleles when virulence was inherited recessively or additively. If virulence were inherited additively, fitness costs decreased the frequency of virulence. Combined, these results suggest that a refuge may prevent virulent *A*. *glycines* biotypes from overcoming *Rag* genes if this aphid-resistance were used commercially in North America.

## Introduction

In 2000, *Aphis glycines* Matsumura was first observed in the US. Prior to 2000, insecticide use in north central US soybean was infrequent [1]; however, after the establishment of *A*. *glycines* insecticide use on soybean in north central US dramatically increased [2]. The reason for the increase in insecticide use is due to soybean yield reductions of up to 40% caused by *A*. *glycines* feeding [3]. Insecticides are effective at reducing *A*. *glycines* populations and preventing associated yield loss while also being cost effective [3, 4]. The insecticides commonly used to manage *A*. *glycines* populations are broad-spectrum and reduce populations of natural enemies present in soybean during application [5, 6, 7]. However, concerns for the future development of insecticide-resistant *A*. *glycines* populations if insecticides are consistently used suggest a need for additional management tools. An alternative strategy that is potentially more cost effective with negligible effects on natural enemies is soybean that contain one or more resistant to *A*. *glycines* genes (or *Rag* genes) [3, 8, 9]. Although there is evidence that *Rag* genes are effective, a limited number of varieties containing *Rag1*, *Rag2*, or *Rag1+Rag2* are commercially available and their adoption is limited [8, 9, 10].

Initially the low adoption of *Rag* soybean was attributed to the concern for reduced yields associated with soybean varieties containing *Rag* genes. However, no yield drag is associated with the presence of *Rag1*, *Rag2*, or both genes [11, 12, 13, 14]. A second factor limiting the production and adoption of *A*. *glycines-*resistant soybean is the discovery of virulent (*i*.*e*., able to feed on soybean containing aphid-resistance genes) *A*. *glycines* biotypes [15, 16, 17]. To date four biotypes have been confirmed in North America [15, 16, 17]. Biotype-1 is avirulent to all of the *Rag* genes currently known. Biotype-2 is virulent towards the *Rag1* gene [15]. Biotype-3 is virulent towards the *Rag2* gene [16], and biotype-4 is virulent towards both the *Rag1* and *Rag2* genes, as well as pyramids constructed with both *Rag1* and *Rag2* [17].

The occurrence of virulence in North America is disconcerting, as their presence may limit the durability of aphid-resistance as a management tool if the use of *Rag* genes becomes more common. However, insect resistance management (IRM) programs that incorporate a refuge of susceptible plants can limit the frequency of virulent biotypes, but their utility is limited based on the biology and life-history traits of the target pest [18]. For example, a refuge has limited value in reducing the increase of a virulent insect that reproduces parthenogenetically, like *A*. *glycines*. However, when virulence is associated with a reduction in fitness on a susceptible host plant then a refuge can delay an increase in virulence. Gassmann et al. [19] define fitness costs as trade-offs in which alleles that confer higher fitness in one environment (*e*.*g*., on *Rag* soybean) reduce fitness in an alternative environment (*e*.*g*., on an aphid-susceptible soybean). Fitness costs in a virulent population result in a reduction of the frequency of virulent alleles when refuges of susceptible plants are present [20]. In addition to fitness costs, negative cross-resistance can also reduce the frequency of virulence in a population. Negative cross-resistance occurs when the allele(s) that confer virulence to one resistance gene also confer hypersensitivity to another resistance gene. An alternative to negative cross-resistance is negatively correlated resistance, which occurs when the gene responsible for virulence to one source of resistance is not the same gene that is responsible for hypersensitivity to another source of resistance [20]. Crowder and Carrière [18] determined that a resistant crop for parthenogenic insects would only be effective for a short period of time unless fitness costs were associated with virulence.

To date, only two studies have evaluated the fitness of a single virulent biotype, biotype-3. Wenger et al. [21] evaluated the fitness costs associated with virulence to *Rag2* (*i*.*e*., biotype-3’s fitness on susceptible soybean), while Enders et al. [22] examined biotype-3 for negative cross-resistance to *Rag1*. Wenger et al. [21] observed fitness costs for biotype-3 on susceptible soybean, and also concluded that virulence is not complete. Enders et al. [22] observed negative cross-resistance for biotype-3 on *Rag1* soybean. Therefore, it is possible that the presence of these factors in virulent *A*. *glycines* biotypes could affect the rate at which virulence alleles increase in the environment. If either a fitness cost or negative cross-resistance occur within *A*. *glycines*, the impact could reduce the perceived importance of virulence as a hindrance to the successful and sustainable adoption of *A*. *glycines*-resistant soybean.

The objective of this study was to determine if fitness costs or negative cross-resistance are associated with virulence to *Rag1* or *Rag2* for *A*. *glycines* biotype-1, biotype-2, biotype-3, and biotype-4 populations on near-isogenic resistant and susceptible soybean cultivars. In addition to this evaluation, we sought to determine if *A*. *glycines* biotype-1 could obviate a fitness cost associated with virulence to *Rag1* or *Rag2* [23]. Finally we used a deterministic genetic model to predict the relative frequency of virulent *A*. *glycines* in light of fitness costs and negative cross-resistance observed herein.

## Materials and Methods

### Aphid colonies and soybean cultivars

*Aphis glycines* populations used for this experiment were obtained from The Ohio State University and the University of Wisconsin. Four populations that were defined by their response to *Rag1* and *Rag2* genes were utilized. A biotype avirulent to *Rag1* and *Rag2* (biotype-1; The Ohio State University), a biotype virulent to *Rag1* but not *Rag2* (biotype-2; The Ohio State University), a biotype virulent towards *Rag2* but not *Rag1* (biotype-3; The Ohio State University), and a biotype virulent towards *Rag1* and *Rag2* (biotype-4; University of Wisconsin) [11, 16, and 17]. These populations were initially collected and identified in Ohio and Wisconsin using detached leaf assays (described in [24]). Biotype-1 *A*. *glycines* were reared and maintained on susceptible soybean, biotype-2 *A*. *glycines* reared and maintained on *Rag1* soybean, biotype-3 *A*. *glycines* reared and maintained on *Rag2* soybean, and biotype-4 *A*. *glycines* reared and maintained on *Rag1+Rag2* soybean. Cultivars used for rearing and maintaining the aphids are near-isogenic (≥ 75% of genes from the recurrent parent IA3027). Soybean plants used contained either no *Rag* genes (IA3027), *Rag1* (IA3027RA1), or *Rag1*+*Rag2* (IA3027RA12) and are near-isolines for the resistance genes *Rag1* and *Rag2* (approximately 93.75% genetically identical) [12, 13]. The near-isogenic line containing only the *Rag2* gene is an experimental soybean line with 75% of its genes derived from the recurrent parent line IA3027 [25].

### Fitness costs associated with virulence of biotype-2 and biotype-3 on susceptible soybean

We hypothesized that the fitness of biotype-2 and biotype-3 *A*. *glycines* would be lower on susceptible soybean, when compared to biotype-1 (*i*.*e*., fitness costs would be associated with virulence to *Rag1* or *Rag2*). We estimated the fitness of each biotype on *Rag1*, *Rag2*, and susceptible soybean by infesting plants with five mixed age apterous *A*. *glycines* of either biotype-1, biotype-2, or biotype-3, and measuring the population density on the plant 11 d after infestation. Fitness costs were identified if the population densities of the virulent biotype-2 and biotype-3 *A*. *glycines* were significantly lower than that of biotype-1 on the susceptible soybean. In addition, negative cross resistance was identified if the population density of a virulent biotype was significantly lower than that of avirulent biotype on soybean containing a resistance gene that it is not virulent towards. For example, if biotype-3 (virulent to *Rag2*, but avirulent to *Rag1*) has fewer aphids after 11 days on a *Rag1* variety than biotype-1 (avirulent) then we would conclude that biotype-3 has negative cross-resistance. In total, we used nine treatments; each treatment was a combination of two factors, soybean cultivar (3 levels) and *A*. *glycines* biotype (3 levels).

*Aphis glycines* individuals were transferred from colonies maintained at Iowa State University to the first full trifoliate of individually potted plants at the second trifoliate growth stage (V2 according to [26]). Plants were enclosed within mesh nets to prevent plant-to-plant movement of aphids. After 24 h we examined *A*. *glycines* populations to confirm their successful establishment. The total number of *A*. *glycines* (both nymphs and adults) present on each plant was counted 11 d after initial infestation.

This experiment was repeated twice using a randomized complete block design (RCBD) with five blocks per repetition (10 experimental units per treatment). Individually potted soybean plants were grown in 16-cm diameter pots in a growth chamber (E41L2C9, Percival Scientific, Incorporated, Perry, IA) using a 14:10 light:dark cycle and a constant temperature of 27°C with a relative humidity of 60%.

### Fitness cost associated with virulence of biotype-4 on susceptible soybean

Biotype-4 was not included in the previous experiment because a colony had not been established at the onset of that experiment. We next hypothesized that *A*. *glycines* biotype-4 fitness would be lower on susceptible soybean when compared to biotype-1 (*i*.*e*., fitness costs would be present). We tested for fitness costs using the same experimental procedures as described previously. Plants were infested with biotype-1 or biotype-4, and the population density was measured 11 d after initial infestation.

We utilized a two factor experimental design, with eight total treatments. Each treatment was a combination of four soybean cultivars and two *A*. *glycines* biotypes. Three of the soybean cultivars used were the same as described in the previous experiment, with the addition of a resistant cultivar containing *Rag1*+*Rag2* (IA3027RA12). Each cultivar was infested with either five biotype-1 or five biotype-4. The fitness of these two biotypes on each soybean cultivar was measured in population density and compared to determine the presence of fitness costs. The method for infesting *A*. *glycines* populations from the previous experiment was used. The same planting procedure and growth chamber specifications as the previous experiment were used. This experiment was repeated twice using a RCBD with five blocks within each repetition within each repetition (10 experimental units per treatment).

### Impact of induced susceptibility on fitness costs of biotype-2 and biotype-3 on susceptible soybean

Varenhorst et al. [23] observed obviation of *Rag* resistance by an improvement in fitness of avirulent A. glycines when the plant is co-infested by virulent biotypes. Our third hypothesis was that the herbivory by biotype-1 would improve the quality of susceptible soybean for biotype-2 and biotype-3. We tested this hypothesis using the same experimental design as outlined by Varenhorst et al. [23]. This design involved infesting soybean plants with an initial population (referred to as the inducer population) for 24 h prior to infesting plants with a second population (referred to as the response population). The population density of the response population 11 d after infestation is a measurement of the effect of the inducer population. The effect of the inducer population on the performance of the response population can then be assessed in comparison to plants receiving a response population but not an initial inducer population. If response populations were greater in the presence of a biotype-1 inducer population than in their absence, then we infer that induced susceptibility alleviates fitness costs.

For this experiment, we used a three-factor design with ten total treatments. The three factors included plant cultivar, inducer population biotype, and response population biotype. We utilized three soybean cultivars: susceptible, *Rag1*, and *Rag2*. Four inducer populations were used: no inducer (none), 50 biotype-1 (B1), 50 biotype-2 (B2), or 50 biotype-3 (B3). Three response populations were used: five biotype-1, five biotype-2, or five biotype-3. Inducer populations were applied to the first full trifoliate when the plants reached the second trifoliate growth stage. The entire first trifoliate was then enclosed within a mesh net. The inducer populations remained on the plans for the duration of the experiments. For plants that did not receive an inducer population, the first trifoliate was caged but no aphids were added. After 24 h, the response populations were added to the second full trifoliate and allowed to move freely about the plant, with the exception of the first trifoliate, which was enclosed in a net that contained the inducer population. The mesh nets are effective at separating the inducer and response populations [23]. Each potted plants was enclosed within mesh nets to prevent plant-to-plant movement. Response populations were examined after 24 h to confirm successful establishment. The total number of *A*. *glycines* present in the response population on each plant was counted 11 d after the initial infestation. The experiment was repeated twice using a RCBD with three blocks per repetition (six total experimental units per treatment). The same planting procedure and growth chamber specifications as the previous experiments were used.

### Statistical analysis

To address each hypothesis, we analyzed the number of *A*. *glycines* per plant after 11 d. The *A*. *glycines* per plant data were log transformed to reduce heteroscadacity. All data for the first two experiments were analyzed using the PROC MIXED procedure with SAS statistical software version 9.3 (SAS Institute, Cary, NC). For both experiments, data were analyzed using an analysis of variance (ANOVA). Significant treatment effects were then separated using *F-*protected least-squares means with a significance level of *P <* 0.05.

The statistical model used to analyze data from the first two experiments (*i*.*e*., fitness costs of biotype-2 and biotype-3, and fitness costs of biotype-4) included the main effects of repetition, block, soybean cultivar, and *A*. *glycines* biotype. All two and three-way interactions of the main effects were included in the model.

All data from the third experiment (*i*.*e*., biotype-1 obviation of fitness costs on susceptible soybean) were analyzed using the PROC GLM procedure. Data were analyzed using an analysis of variance (ANOVA) with significant treatment effects separated using Student-Newman-Keuls (SNK) grouping with a significance level of *P <* 0.05. The statistical model included the main effects of repetition, block, inducer population biotype, and response population biotype. All two- and three-way interaction terms among the main effects were included in the model.

### Modeling the consequences of fitness costs and negative cross-resistance

We hypothesized that the fitness cost and negative cross-resistance we observed would affect the rate at which virulence alleles increased within a population of *A*. *glycines*. We utilized a simple deterministic, single-locus, four compartment genetic model developed for *A*. *glycines* to track changes in the frequency of virulence alleles [23]. The model was adapted from one created for parthenogenic reproducing insects similar to *A*. *glycines* [18]. We tracked the change in the frequency of virulence alleles across 25 years with 14 generations of asexual reproduction and one generation of sexual reproduction occurring within each year. We assumed virulence to the *Rag1* and *Rag2* genes to be conferred by two independently segregating genes. Each virulence gene was assumed to have two alleles, with one allele conferring virulence and another conferring avirulence. Mating was assumed to be completely random with alleles returning to Hardy-Weinberg equilibrium after each year’s generation of sexual reproduction. We tracked the frequency of virulence alleles to *Rag1* for 25 years beginning at the initial deployment of resistant cultivars. We report the number of years for the frequency of the *Rag1* virulence allele to surpass 50% in the population. We report the allele frequency after 25 years for the *Rag1* virulence allele in cases where the frequency fails to surpass 50% in 25 years.

Our goal was to evaluate the relative potential importance of fitness costs and negative cross-resistance for the development of virulence, not to evaluate all possible scenarios for the development of virulence to *Rag* genes. Therefore, we assessed a small proportion of possible scenarios for the development of virulence in *A*. *glycines*. We used values from empirical data for specific parameters in the *A*. *glycines-*soybean system whenever possible, including the field-to-field movement rate of *A*. *glycines* (5% static rate, [27]), the efficacy of the *Rag1* and *Rag2* genes (41% reduction in *A*. *glycines* fitness, [28]), the efficacy of a *Rag1+Rag2* pyramid (59% reduction in *A*. *glycines* fitness, [28]).

Fitness costs and negative cross-resistance were included as reductions in the fitness of virulent individuals on susceptible and resistant plants, respectively. The exact values used for the reduction in fitness due to fitness costs and negative cross-resistance were based on the results of experiments one and two. We included induced susceptibility effects in all of our models [23] as these can affect the rate at which virulence alleles increase in the population. The inclusion of a density-dependent increase in fitness was used to model induced susceptibility. A starting population density of 1.0×10^2^ individuals per 1% of the landscape was used for each year of the model. Fitness of individuals in a compartment increased to 1.65 if the population density surpassed 1.0×10^13^ individuals per 1% of landscape. Obviation of resistance was modeled by setting the fitness of all individuals in a resistant compartment to 1.0 when the population density of homozygous virulent individuals surpassed 1.0×10^13^ per 1% of landscape. Obviation of fitness costs was modeled using the same method as obviation of resistance, except the obviation of fitness costs was based on the population density of homozygous avirulent individuals in the susceptible compartment of the landscape.

We ran the model for a range of values for unknown parameters, including the dominance of virulence (recessive, additive, and dominant), and initial virulence allele frequency (0.02 and 0.2, [29]). We assumed a single static 25% refuge size of susceptible plants in the landscape for each run of the model. We investigated two resistance gene deployment strategies by partitioning the remaining 75% of the landscape to either plants containing only the *Rag1* gene or plants containing the *Rag1*+*Rag2* genes. In total, we ran the model 24 times once with fitness costs and negative cross-resistance included and once without for each combination of dominance of virulence (three levels), initial allele frequency (two levels), and gene deployment strategy (two levels).

## Results

### Fitness costs associated with virulence of biotype-2 and biotype-3 on susceptible soybean

We confirmed our hypothesis that the population densities of *A*. *glycines* biotype-2 and biotype-3 would be lower on susceptible soybean when compared to biotype-1 (*i*.*e*., a fitness costs associated with virulence). This was observed by analyzing data for the significance of the main effects of soybean cultivar, *A*. *glycines* biotype, and the interaction of soybean cultivar by *A*. *glycines* biotype. The population densities of the biotypes of *A*. *glycines* varied significantly by the main effects of soybean cultivar (*F* = 14.41; df = 2, 76; *P <* 0.0001), *A*. *glycines* biotype (*F* = 5.83; df = 2, 76; *P <* 0.0044), and their interaction (*F* = 58.00; df = 4, 76; *P <* 0.0001). We observed unequal population densities of the biotypes among the soybean cultivars, therefore we analyzed these data by soybean cultivar.

**Fig 1:**
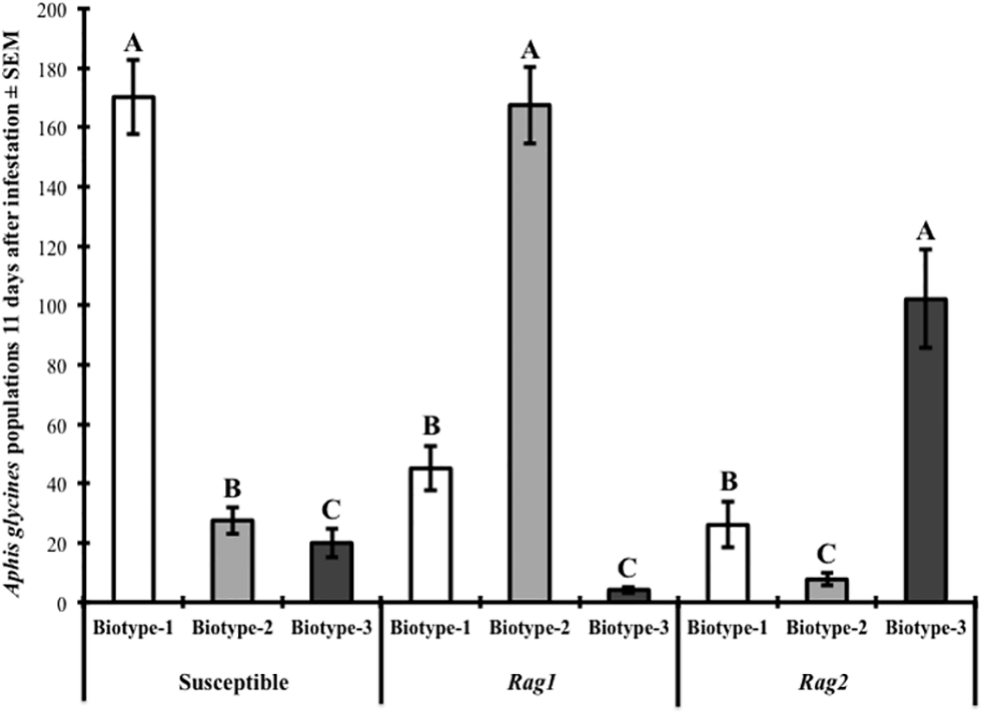
Biotype-2 and biotype-3 *A*. *glycines* reproduction reduced on a susceptible soybean compared to aphid resistant cultivars. Note that biotype 1 is avirulent to both aphid-resistant cultivars, but virulent to the susceptible cultivar. The susceptible soybean cultivar was IA3027, the *Rag1* cultivar was IA3027RA1, and the *Rag2* cultivar was IA3027RA2. Data were analyzed by soybean cultivar, and capital letters indicate significance differences across biotypes (*P <* 0.05).

On susceptible soybean, the population density of *A*. *glycines* biotype-1 was significantly greater than those of biotype-2 (*t* = 4.13; df = 2, 22; *P <* 0.0004) and biotype-3 (*t* = 6.80; df = 2, 22; *P <* 0.001) (Fig 1). The population density of biotype-2 was also significantly greater than that of biotype-3 (*t* = 2.68; df = 2, 22; *P <* 0.0138) on susceptible soybean (Fig 1). For the *Rag1* soybean cultivar, the population density of biotype-2 was significantly greater than those for biotype-1 (*t* = 6.72; df = 2, 22; *P <* 0.0001) or biotype-3 (*t* = 11.76; df = 2, 22; *P <* 0.0001) (Fig 1). The population density of biotype-1 was also significantly greater than that of biotype-3 (*t* = 5.04; df = 2, 22; *P <* 0.0001) on *Rag1* soybean. On *Rag2* soybean, the population density of biotype-3 was significantly greater than those of biotype-1 (*t* = 5.15; df = 2, 22; *P <* 0.0001) or biotype-2 (*t* = 8.68; df = 2, 22; *P <* 0.0001). The population density of biotype-1 was also significantly greater than that of biotype-2 (*t* = 3.53; df = 2, 22; *P <* 0.0019) on *Rag2* soybean (Fig 1).

In summary, these results indicate that for the susceptible soybean cultivar tested, the population densities of both biotype-2 and biotype-3 were lower than those of biotype-1. In addition, these results indicate that population densities of biotype-2 and biotype-3 were lower on *Rag2* and *Rag1* respectively when compared to biotype-1.

### Fitness cost associated with virulence of biotype-4 on susceptible soybean

In our second experiment, we confirmed our hypothesis that the population density of *A*. *glycines* biotype-4 would be lower on susceptible soybean when compared to biotype-1 (*i*.*e*., fitness costs for this biotype exist on susceptible soybean). As in the first experiment, data were analyzed for the significance of the main effects of soybean cultivar, *A*. *glycines* biotype, and the interaction of soybean cultivar by *A*. *glycines* biotype. The population densities of the biotypes of *A*. *glycines* varied significantly by the main effects of soybean cultivar (*F* = 50.31; df = 3, 67; *P <* 0.0001) and *A*. *glycines* biotype (*F* = 138.00; df = 1, 67; *P <* 0.0001). The interaction of soybean cultivar by *A*. *glycines* biotype was significant (*F* = 98.36; df = 3, 67; *P <* 0.0001), and indicated that the two *A*. *glycines* biotypes did not respond similarly to the soybean cultivars. Similar to the previous experiment we observed unequal reduction in the populations of the *A*. *glycines* biotypes among the soybean cultivars, therefore data were analyzed by soybean cultivar.

**Fig 2:**
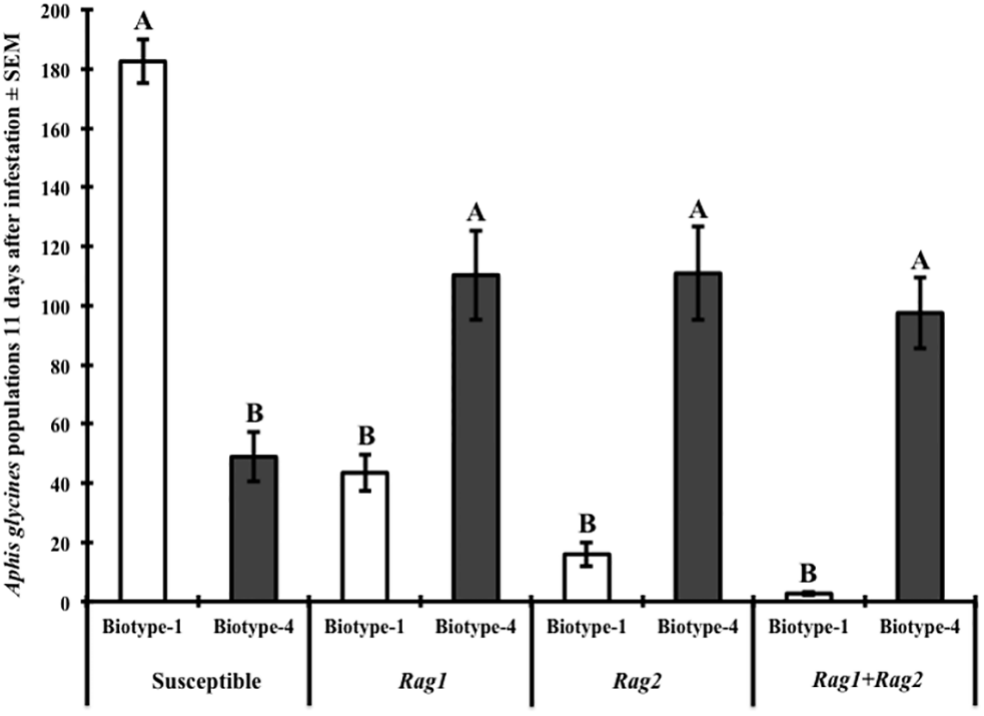
Biotype-4 *A*. *glycines* reproduction reduced on a susceptible soybean compared to an aphid resistant cultivar. Note that biotype 1 is avirulent to both aphid-resistant cultivars, but virulent to the susceptible cultivar. For this experiment the susceptible soybean cultivar was IA3027, the *Rag1* cultivar was IA3027RA1, the *Rag2* cultivar was IA3027RA2, and the *Rag1*+*Rag2* cultivar was IA3027RA12. Data were analyzed by soybean cultivar, and capital letters indicate significance differences between biotype populations (*P <* 0.05).

On susceptible soybean, the population density of *A*. *glycines* biotype-1 was significantly greater than that of biotype-4 (*t* = 6.91; df = 1, 13; *P <* 0.0001) (Fig 2). For the *Rag1* (*t* = 4.19; df = 1, 13; *P <* 0.0011), *Rag2* (*t* = 9.29; df = 1, 13; *P <* 0.0001), and the *Rag1*+*Rag2* (*t* = 18.74; df = 1, 13; *P <* 0.0001) soybean cultivars, the population density of biotype-4 was significantly greater than that of biotype-1 (Fig 2). These results indicate that for the susceptible soybean cultivar that was tested, the population density of biotype-4 was lower on susceptible soybean when compared to biotype-1.

### Impact of induced susceptibility on fitness costs of biotype-2 and biotype-3 on susceptible soybean

**Fig 3:**
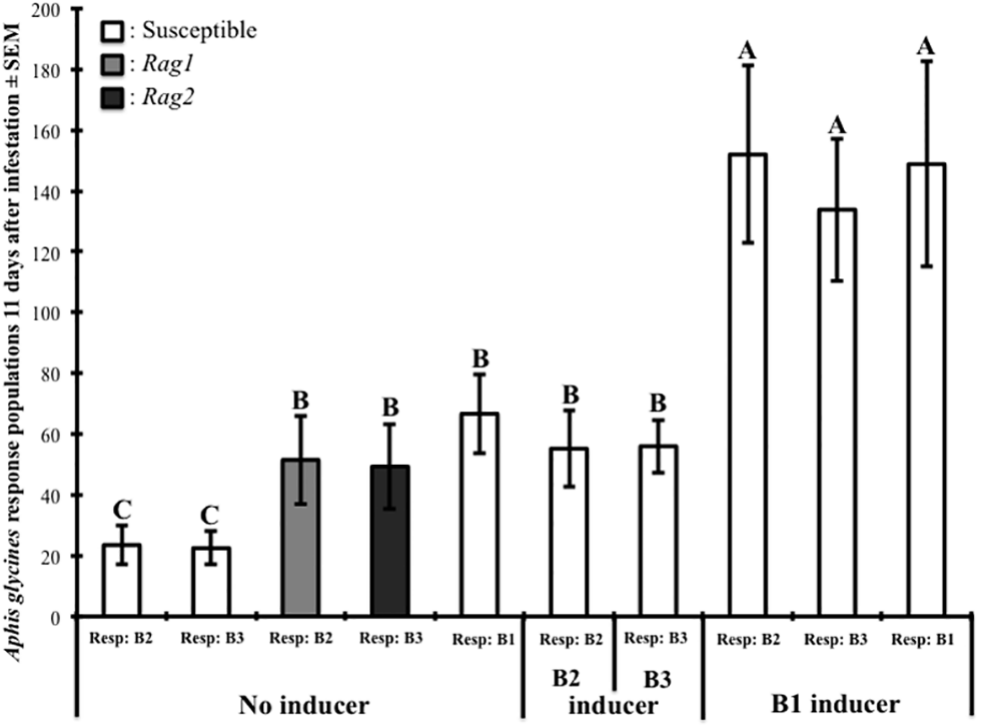
Addition of an inducer population affects subsequent fitness of virulent biotypes on a susceptible cultivar. This effect was observed by varying inducer population biotypes on varying response population biotypes on susceptible soybean. For this experiment the susceptible cultivar IA3027, *Rag1* cultivar IA3027RA1, and *Rag2* cultivar IA3027RA2 were used. Capital letters indicate significance among treatments (*P <* 0.05).

For our third experiment, we confirmed that *A*. *glycines* biotype-1 is capable of alleviating the fitness costs observed for biotype-2 and biotype-3 on the susceptible soybean tested. To test this hypothesis, we analyzed these data for the significance of the main effects of inducer population biotype, response population biotype, and the interaction of inducer population biotype by response population biotype. The main effects of inducer population biotype (*F* = 31.05; df = 1, 41; *P <* 0.0001) and response population biotype (*F* = 28.83; df = 2, 41; *P <* 0.0001) both significantly affected the density of the response population. The interaction of inducer population biotype by response population biotype was also significant (*F* = 5.06; df = 2, 41; *P <* 0.0209). In agreement with results from Varenhorst et al. [23], both the presence and herbivory of biotype-1 positively affected the density of the biotype-2 and biotype-3 response populations on susceptible soybean (Fig 3).

When inducer populations were absent the biotype-1 response population was 184% greater than biotype-2 and 196% greater than biotype-3. The biotype-1 response population without an inducer population was 21% greater than biotype-2 and 19% greater than biotype-3 when both had their respective biotype inducer populations present. In the presence of the biotype-1 inducer population, there were no significant differences among the population densities of biotype-1, biotype-2, or biotype-3 response populations (Fig 3). In summary, the differences between the population densities of avirulent and virulent biotypes on susceptible soybean were diminished with the addition of an inducer population with the same biotype.

### Modeling the consequences of virulent biotype fitness costs and induced susceptibility

In general, fitness costs and negative cross-resistance delayed the development of virulence. This delay was observed when virulence was rare (*i*.*e*., 0.02), regardless of the mode of inheritance. When virulence was common (*i*.*e*., 0.2), the delay was only observed with recessive or additive modes of inheritance (Table 1). Fitness costs resulted in the frequency of additively inherited virulence alleles to actually decrease over the course of 25 years. Dominantly inherited virulence alleles were only slightly delayed by the presence of fitness costs.

**Table 1:** Simulated effect of induced susceptibility and fitness costs on virulence development.

Model Factors Included	Default^a^		Default Including Fitness Costs	
Initial Vir. Allele Frequency	0.02	0.2	0.02	0.2
**Recessive** ^**b**^				
*Rag1* Alone	>25^c^ (0.02)^d^	4	>25 (0.02)	18
*Rag1+Rag2* Pyramid	>25 (0.02)	25	>25 (0.02)	>25 (0.03)
**Additive**				
*Rag1* Alone	>25 (0.03)	4	>25 (<0.01)	>25 (<0.01)
*Rag1+Rag2* Pyramid	>25 (0.02)	14	>25 (<0.01)	>25 (<0.01)
**Dominant**				
*Rag1* Alone	>25 (0.06)	3	>25 (0.03)	3
*Rag1+Rag2* Pyramid	>25 (0.02)	4	>25 (<0.01)	4

^a^ Default simulations were run with the inclusion of induced susceptibility effects but not fitness costs or negative cross-resistance effects.

^b^ Inheritance of virulence and fitness costs.

^c^ Years until the frequency of the allele conferring virulence to *Rag1* exceeded 0.50.

^d^ If the frequency of the virulence allele failed to surpass 0.50 within 25 years, its frequency after 25 years is provided in parentheses

The decreases in the rate of virulence evolution (*i*.*e*., reduced virulent allele frequency) were due to two factors. First, between generations 10 and 11, the frequency of virulence alleles decreased due to the increased fecundity of individuals in the refuge, as a result of fitness costs for virulent biotypes on susceptible soybean. The fecundity of individuals in the refuge was temporarily higher because the density in that landscape reached 1.0×10^13^ a generation prior to individuals on resistant plants, as a result of obviation of fitness costs. Second, the selection pressure imposed by the resistance gene was alleviated by generation 11 because the density of homozygous resistant individuals exceeded 1.0×10^13^ in the resistant landscape, a result of induced susceptibility.

## Discussion

Our results indicate that fitness costs exist for biotype-2, biotype-3 and biotype-4 *A*. *glycines* on the susceptible soybean cultivar (IA3027) that was used for these experiments when compared to cultivars on which they are virulent. Within our experimental design, we determined that at 11 d the populations of biotype-2 were reduced by 84% (Fig 1), biotype-3 by 88% (Fig 1), and biotype-4 by 73% (Fig 2) compared to biotype-1 on the susceptible soybean. The difference in reproduction between the virulent biotypes and our avirulent biotype on the susceptible soybean can be explained by two general hypotheses. First, there exists some here-to-fore undescribed aphid-resistance within the aphid-susceptible variety used in our experiments to which biotypes-2 through 4 are avirulent, but biotype-1 is virulent. If correct, this would require us to refer to susceptible soybean as “wild type” as this innate aphid-resistance can negatively impact virulent biotypes. Second, biotypes that are virulent towards *Rag* genes identified to-date, are specialized to such a degree that they cannot effectively reproduce on a susceptible soybean. Evidence supporting our second hypothesis is observed by the presence of either negative cross-resistance or negatively correlated resistance of biotype-2 to *Rag2*, and biotype-3 to the *Rag1* soybean cultivars used. These results suggest that the specialization of biotype-2 to feed on *Rag1* and biotype-3 to feed on *Rag2* prevent it from successfully feeding on the alternative *Rag* gene.

In general, salivary secretions (including but not limited to effector proteins) can alter the plants’ physiology making it a better host for the aphid [30, 31]. Although there is no evidence that effector proteins are responsible for virulent aphids in the *A*. *glycines*-soybean system, there is evidence from other aphid-plant systems that effector proteins are responsible for virulence to aphid resistant plants [32]. Furthermore, variation in effector proteins can explain variation in virulence, as noted for *Diuraphis noxia* Kurdjumov [32]. Additional evidence that effector proteins play a similar role in *A*. *glycines* include the discovery of genes coding for effector proteins in other aphid species in *A*. *glycines* [33].

Assuming virulence is due to effector proteins, it is possible that the gene or genes responsible for producing these promote virulent biotypes (*e*.*g*., biotype-2) to feed on resistant soybean (*e*.*g*., *Rag1*) are also responsible for the hypersensitivity to the other resistance genes (*e*.*g*., *Rag2*). A similar effect is observed when effector molecules associated with pathogens elicit either positive or negative responses in their host plant [34, 35]. The expression of effector proteins in *A*. *glycines* is likely unique to each biotype, similar to the Russian wheat aphid [32]. Based on this assumption, biotype-1 (avirulent to both *Rag1* and *Rag2*) would have standard effector protein expression with no modifications for virulence to *Rag1* or *Rag2* soybean. Therefore, biotype-1 effectors would be described as *Rag1* negative and *Rag2* negative (B1: *Rag1*-, *Rag2*-). Because biotype-2 is virulent to *Rag1* the expression of its effector proteins are likely modified for virulence on *Rag1* and are best described as *Rag1* positive, but is avirulent for *Rag2* soybean making it *Rag2* negative (B2: *Rag1*+, *Rag2*-). Biotype-3 is virulent to *Rag2* and the expression of its effector proteins would likely vary from that of biotype-1 or biotype-2 and provide virulence to *Rag2* but is avirulent to *Rag1* (B3: *Rag1*-, *Rag2*+). Biotype-4 has effector proteins that are modified for virulence to both *Rag1* and *Rag2* genes (B4: *Rag1*+, *Rag2*+).

The potential role of effector proteins can help explain how virulent biotypes experience fitness costs on susceptible (*i*.*e*., wild type) soybean and *Rag* genes to which they are not virulent. The effector proteins present in the virulent biotypes may not be effective at altering the physiology of a wild type plant or may actually elicit an increased defense response (*e*.*g*., [35]). Similarly, effector proteins could also explain our observations of negative cross resistance. Virulent biotypes feeding on soybean containing resistant genes for which they do not have the appropriate effector proteins are unable to fully utilize those plants, resulting in negative cross-resistance.

As previously mentioned, only two studies of *A*. *glycines* biotypes have addressed fitness costs, and both of those manuscripts addressed fitness costs associated with biotype-3. We note that the fitness cost observed by Enders et al. [22] is in agreement with our results that biotype-3 populations decrease significantly on *Rag1* soybean. Our results are also in agreement with those from Wenger et al. [21], that a fitness cost for biotype-3 was observed on susceptible soybean. Although a different soybean cultivar was used by Wenger et al. [21] for their susceptible plant (SD-0176R), it performed similarly to the one used in our current study (IA3027).

Several reports of virulent biotypes in North America [15, 16, 17] did not observe a fitness cost associated with this virulence. Differences in experimental methodology between our study and those may explain why fitness costs were not previously observed. Previous studies used different, susceptible soybean lines (some near-isogenic, others not genetically related) from one another, densities of *A*. *glycines* for the initial infestation, and the temperatures at which infested plants were maintained. All of these factors can influence the reproduction of *A*. *glycines* [3]. For example, reproduction of *A*. *glycines* is positively influenced by population density of the initial population of aphids [23]. Sufficiently large initial populations can induce susceptibility such that the fitness of subsequent colonizers is improved. If a sufficiently large enough initial population were used this may affect the outcome of the experiments, reducing the likelihood of observing fitness costs. The optimal temperature for *A*. *glycines* development is estimated to be 27.8°C [36], but temperatures used for biotype determination experiments varied from 20–27°C. The variation among temperatures used for these experiments could be a possible explanation for the differences in results. Furthermore, there may be interactions between abiotic factors, like temperature, on the impact of *Rag* genes [37, 38]. However, the extent that the protection from all *Rag* genes is influenced by temperature is unknown [39]. Given these sources of variation in methodology, it is difficult to compare across studies. In the future, we propose a standard for *A*. *glycines* laboratory and greenhouse bioassays in which biotype identity is characterized. We propose that a constant temperature of 27.8 ± 1°C be used for future laboratory experiments with *A*. *glycines*. In addition, we suggest that initial infestations of *A*. *glycines* should be limited to five individuals (adults and nymphs) per plant, on early vegetative stage soybean. The proposed method would potentially reduce the variation observed among these studies.

Without a fitness cost associated with virulence for parthenogenic insects, the frequency of virulence is predicted to increase dramatically even with a refuge [18]. We expanded their genetic model to include fitness costs and negative cross-resistance, and observed a large delay or even the prevention of the evolution of virulence. However, the relative inheritance or expression of virulence (recessive, additive or dominant) had a large impact on both the rate at which virulence evolved and the relative importance of fitness costs. Our modeling efforts highlight two important considerations regarding the *A*. *glycines*- resistant soybean system. First, the discovery of virulent *A*. *glycines* biotypes in the US is not an insurmountable obstacle to the sustainable use of *Rag* genes. Second, the relative inheritance or expression of virulence in each biotype must be determined before an accurate assessment can be made of the durability of *Rag* genes. In conclusion, our results suggest that fitness costs associated with virulence to *Rag* genes allow for a decline in the risk of virulence spreading if an IRM plan is employed. Such a plan could employ a refuge of susceptible soybeans. As Wenger et al. [21] proposed, the use of an interspersed refuge would likely be most efficient and effective for *A*. *glycines*. Development of pyramids with additional resistance genes could be a necessary step in the development of a sustainable gene deployment strategy for *Rag* genes.
